# Supplementary material for: Antigen-specific Fab profiling achieves molecular-resolution analysis of human autoantibody repertoires in rheumatoid arthritis
Source: Nat Commun. 2024 Apr 10;15:3114. doi: 10.1038/s41467-024-47337-x (PMC11006680; doi:10.1038/s41467-024-47337-x)
Supplement: Supplementary file 1 — Supplementary Figs. and Tables [file 41467_2024_47337_MOESM1_ESM.pdf]

Supplementary Figures and Tables

Antigen-specific Fab profiling achieves molecular-resolution analysis of human autoantibody repertoires in rheumatoid arthritis

Eva Maria Stork[1,#], Danique M.H. van Rijswijck[2,3,#], Karin A. van Schie[1], Max Hoek[2,3], Theresa Kissel[1], Hans Ulrich Scherer[1], Tom W.J. Huizinga[1], Albert J.R. Heck[2,3,%], Rene E.M. Toes[1,%], Albert Bondt[2,3,%]

[1] Department of Rheumatology, Leiden University Medical Center, Albinusdreef 2, 2333 ZA Leiden, The Netherlands

[2] Biomolecular Mass Spectrometry and Proteomics, Bijvoet Center for Biomolecular Research and Utrecht Institute for Pharmaceutical Sciences, University of Utrecht, Padualaan 8, Utrecht 3584 CH, The Netherlands

[3] Netherlands Proteomics Center, Padualaan 8, Utrecht 3584 CH, the Netherlands

[#] These authors contributed equally

[%] These authors jointly supervised this work

[*] Correspondence to Albert Bondt (a.bondt@uu.nl)

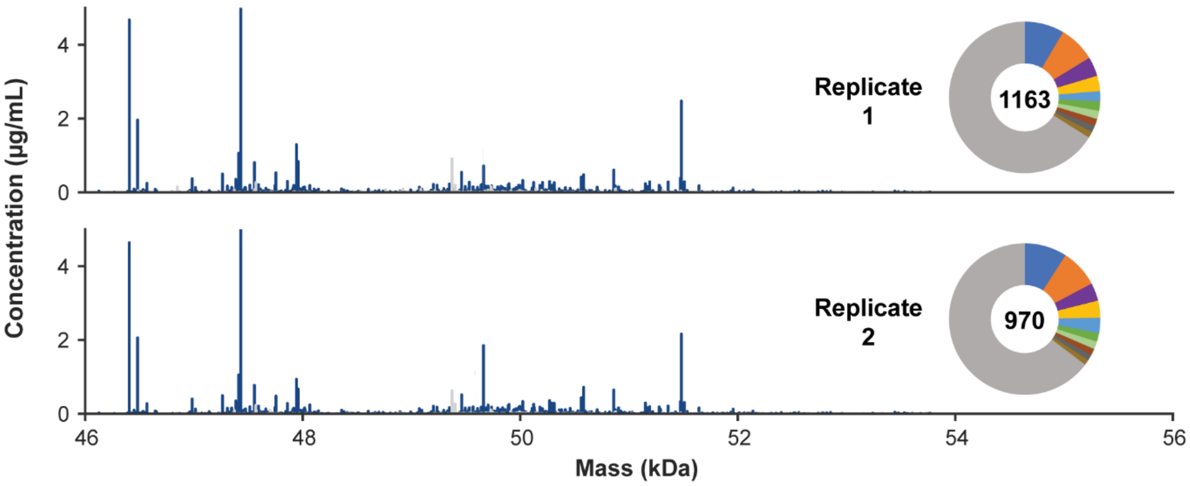

Supplementary Figure 1. Reproducibility of ACPA IgG1 Fab profiling. Reproducibility of ACPA IgG1 Fab profiling was independently replicated by applying a second RA patient plasma n=2 times. ACPA IgG1 Fab profiles of each replicate are shown. The ACPA IgG1 Fab profile of replicate 1 is shown at the top, the ACPA IgG1 Fab profile of replicate 2 is shown at the bottom. Fab molecules shared between both replicates (blue) and unique for the respective profile (grey) are indicated. The relative contribution of the ten most abundant Fab molecules to the total ACPA IgG1 repertoires are depicted as donut plot. Each of the ten most abundant Fab molecules is indicated by a separate color; the remaining Fab molecules are indicated in grey. The total number of unique Fab molecules detected is depicted in the center of each donut plot.

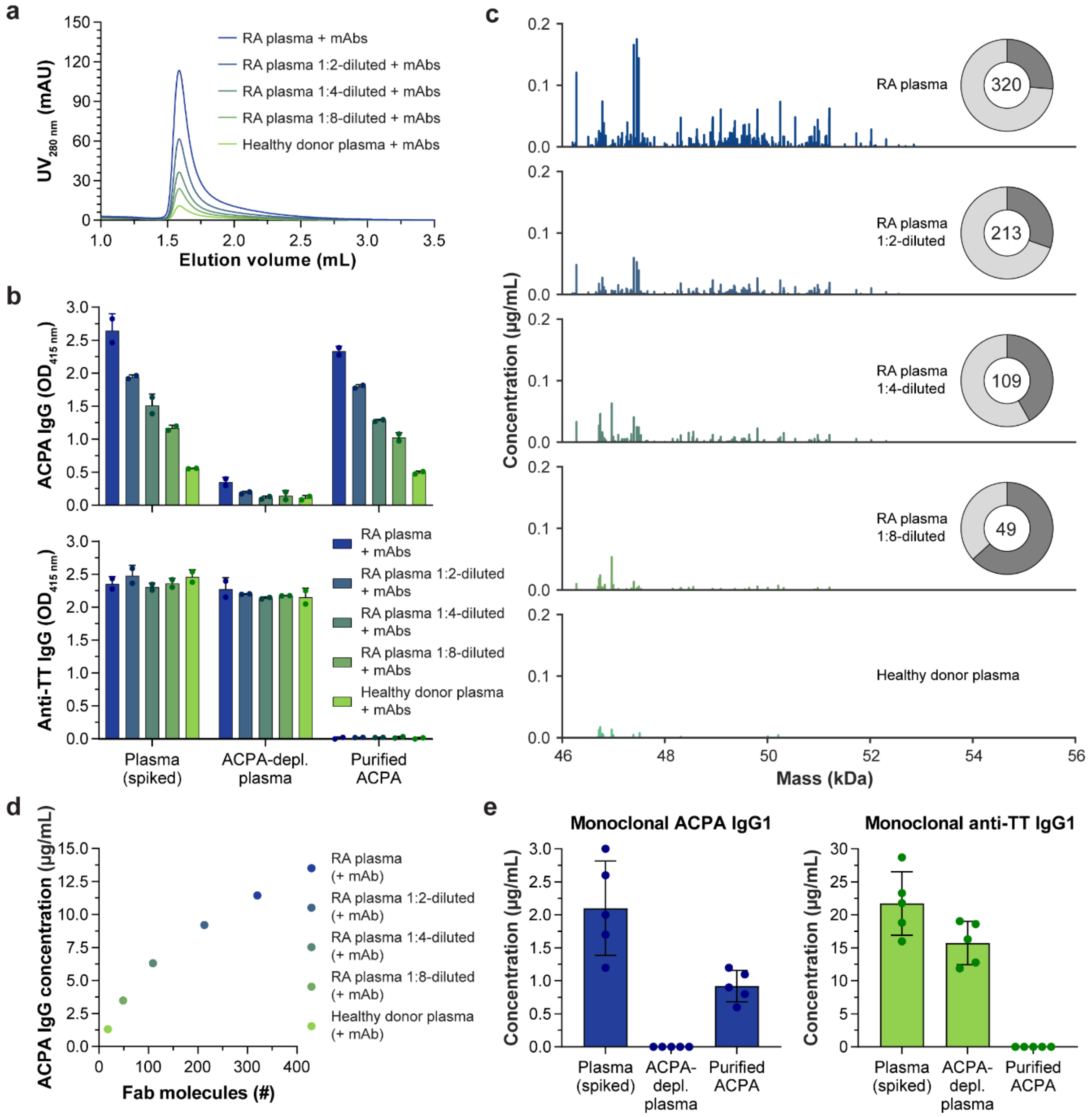

Supplementary Figure 2. Impact of diminishing ACPA levels on measured ACPA IgG1 Fab profiles. The robustness of the ACPA IgG1 Fab profiling approach with respect to varying ACPA levels was assessed by applying RA patient plasma undiluted, 1:2-, 1:4- and 1:8-diluted with a healthy donor plasma (shades of blue to dark green). The undiluted healthy donor plasma was applied as a control (green). The samples were spiked with 20 µg/mL monoclonal anti-TT IgG1 and 2 µg/mL monoclonal non-Fab-glycosylated ACPA IgG1 as internal specificity control (mAbs). **(a)** Chromatograms recorded following elution of ACPA affinity purification, revealing the diminishing amount of ACPAs upon dilution. **(b)** ACPA IgG and anti-TT IgG reactivity of spiked plasma, ACPA-depleted plasma and purified ACPA determined by ELISA. All samples were assessed one time with technical replicates (n=2) at the same dilution. Results are depicted as mean and standard deviation. Individual datapoints are overlaid. Of note, ACPA IgG that was detected in healthy donor plasma and in ACPA purified from healthy donor plasma is caused by the spiked monoclonal ACPA IgG1. **(c)** ACPA IgG1 Fab profiles collected upon subsequent LC-MS-based Fab profiling. Donut plots indicate the relative contribution of the ten most abundant Fab molecules (dark grey) to the total ACPA IgG1 repertoire detected. The total number of unique Fab molecules detected is depicted in the center of each donut plot. Traces caused by the spiked mAbs were removed. **(d)** Correlation of the ACPA IgG concentration determined for each ACPA eluate by total IgG ELISA and the number of unique Fab molecules detected by ACPA IgG1 Fab profiling. Of note, the ACPA IgG concentration includes the spiked monoclonal ACPA IgG1, whereas traces caused by the spiked mAb were removed to determine the number of unique Fab molecules by

ACPA IgG1 Fab profiling. **(e)** Concentrations of monoclonal ACPA and anti-TT IgG1 determined by Fab profiling. Results are depicted as mean and standard deviation. Individual datapoints are overlaid.

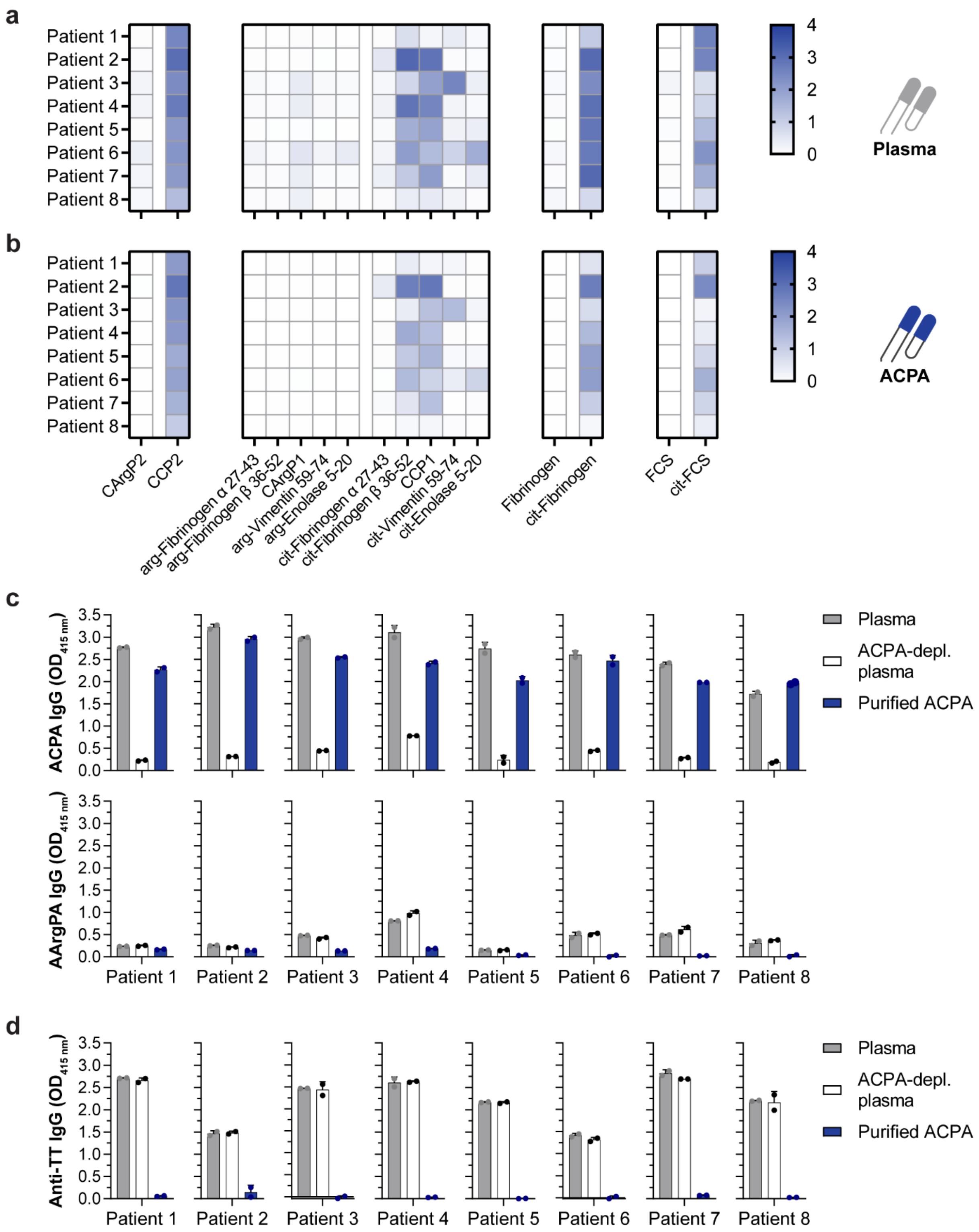

Supplementary Figure 3. ACPA fine-specificity profiles of each plasma in the cohort of RA patients studied (n=8) and monitoring of ACPA purifications. Each patient plasma was characterized for ACPA fine-specificities and the specificity of all ACPA purifications was monitored. **(a)** ACPA fine-specificity profiles determined for each plasma. All plasma were assessed one time at a dilution of 1:100. Binding to each peptide and protein in its modified and non-modified version is depicted as color gradient. **(b-d)** Monitoring of all ACPA purifications by **(b)** ACPA fine-specificity, **(c)** ACPA IgG and **(d)** anti-TT IgG ELISA. IgG binding to CArgP2 (AArgPA IgG) was detected to determine non-citrulline-specific binding to the peptide. ACPA IgG and anti-TT IgG ELISA were performed one time with technical replicates (n=2). Plasma, ACPA-depleted plasma and purified ACPA were generally assessed at the same dilution. Only, for ACPA fine-specificity, ACPA and AArgPA IgG

ELISA, purified ACPA was applied at a dilution corrected for the enrichment of ACPA upon affinity purification. Results are depicted as mean and standard deviation. Individual datapoints are overlaid. Of note, ACPA fine-specificity profiles were largely similar before and after ACPA purification and all purifications were highly specific as anti-TT IgG reactivity was successfully depleted from purified ACPA. ACPA IgG reactivity remaining in ACPA-depleted plasma resembled non-citrulline-specific binding to CArgP2.

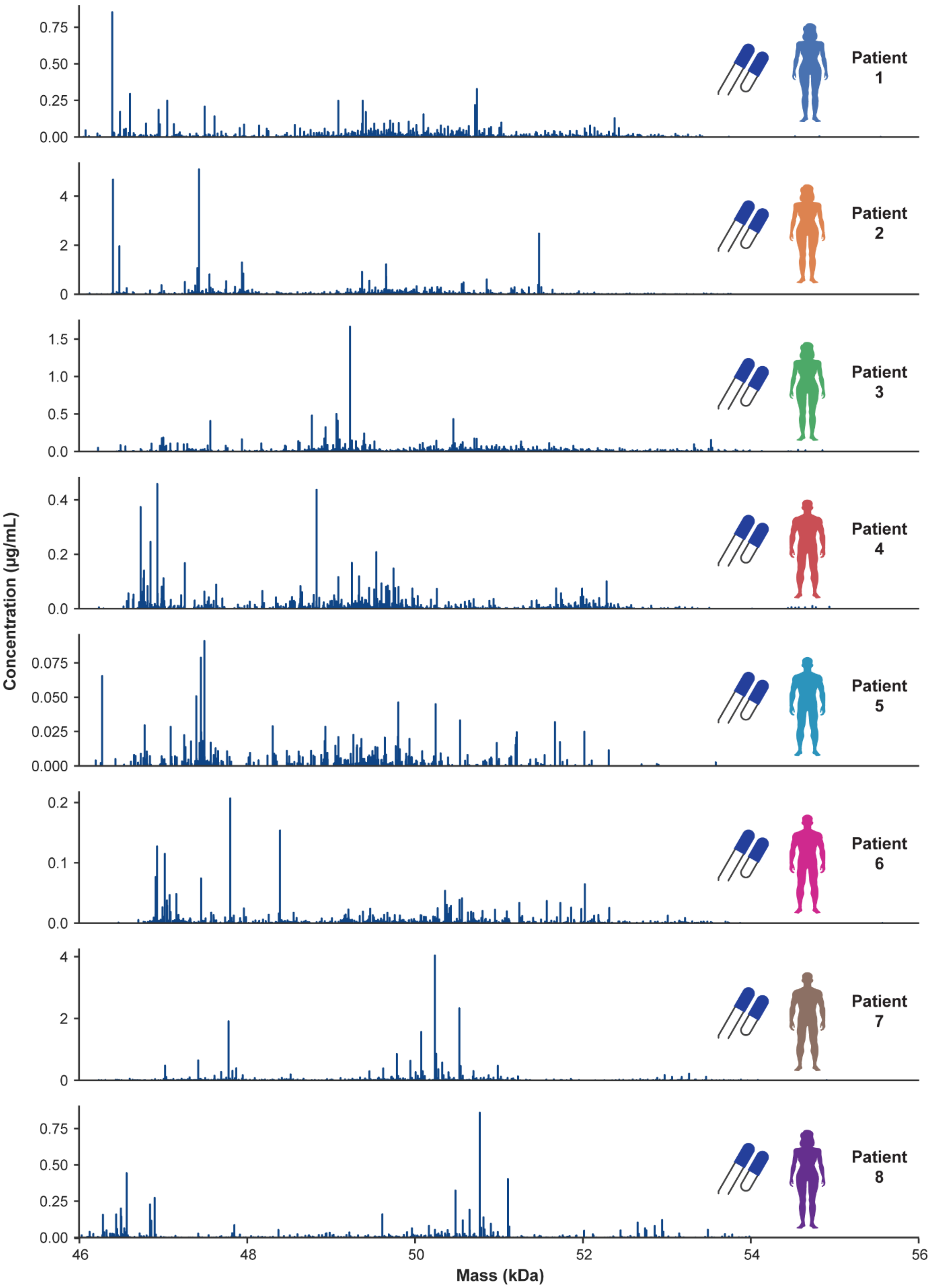

Supplementary Figure 4. ACPA IgG1 Fab profiles of each individual in the cohort of RA patients studied (n=8). Each lane represents the ACPA IgG1 Fab profile obtained for one individual. Y-axes are scaled based on the concentration of the highest abundant Fab molecule of the individual's ACPA IgG1 Fab profile. Of note, plasma of patients 1, 2 and 5 were also used to validate the approach; ACPA IgG1 Fab profiles for patient 1 and 2 were included in determining reproducibility; ACPA IgG1 Fab profiles of patients 2, 3, 4 and 7 are also shown as examples in Figures 2 to 4.

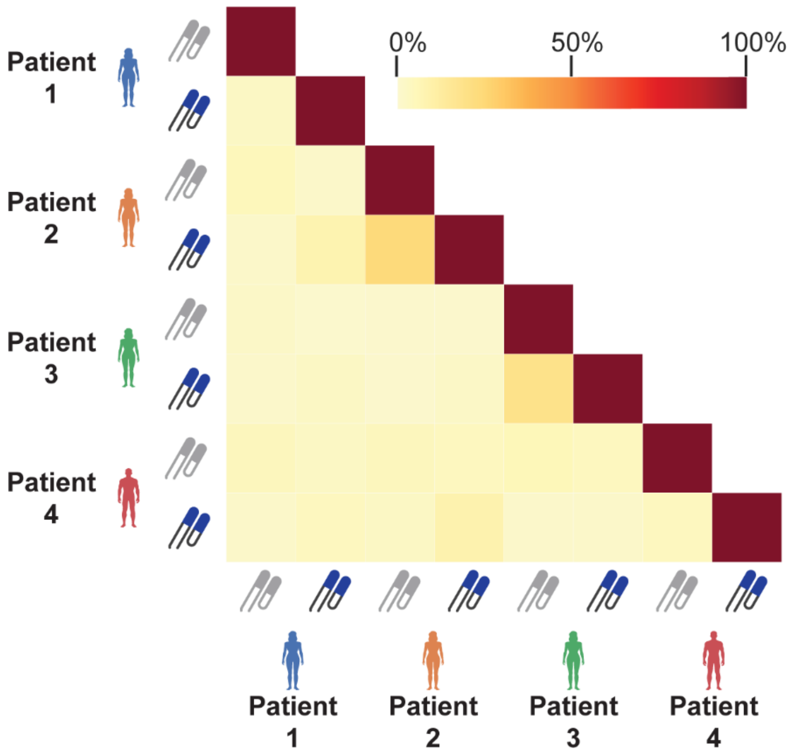

Supplementary Figure 5. Overlap between paired ACPA IgG1 and total plasma IgG1 Fab profiles obtained for n=4 RA patients. ACPA IgG1 and total plasma IgG1 Fab profiles are indicated by blue and grey Fab fragments, respectively. The degree of overlap between Fab profiles is based on the number and abundance of overlapping Fab molecules and indicated by color gradient.

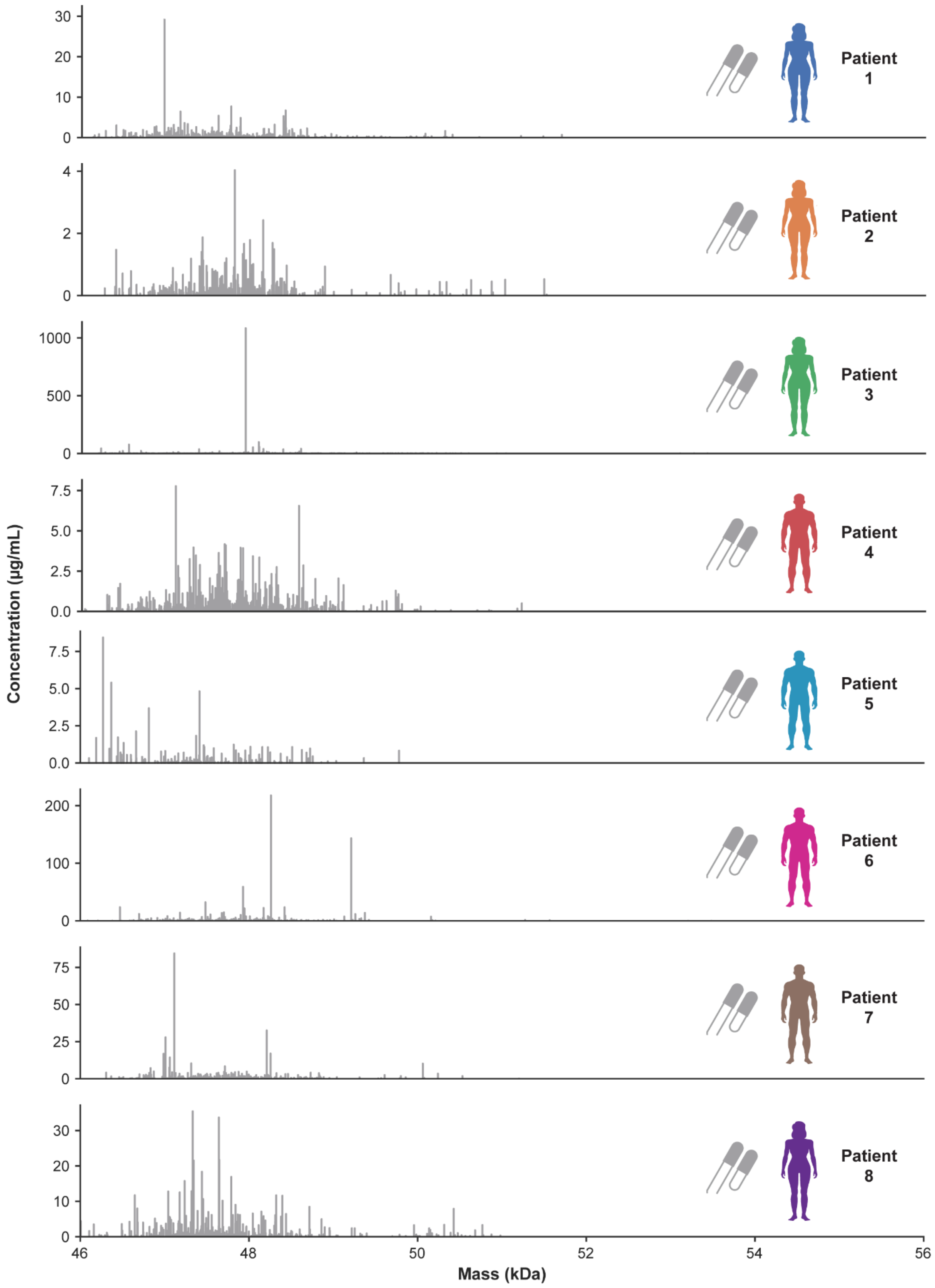

Supplementary Figure 6. Total plasma IgG1 Fab profiles of each individual in the cohort of RA patients studied (n=8). Each lane represents the total plasma IgG1 Fab profile obtained for one individual. Y-axes are scaled based on the concentration of the highest abundant Fab molecule of the individual's ACPA IgG1 Fab profile. Of note, the total plasma IgG1 Fab profile of patient 4 is shown as an example in Figure 3. Besides, note: the difference in the y-axes compared to the paired ACPA IgG1 Fab profiles depicted in Supplementary Figure 4.

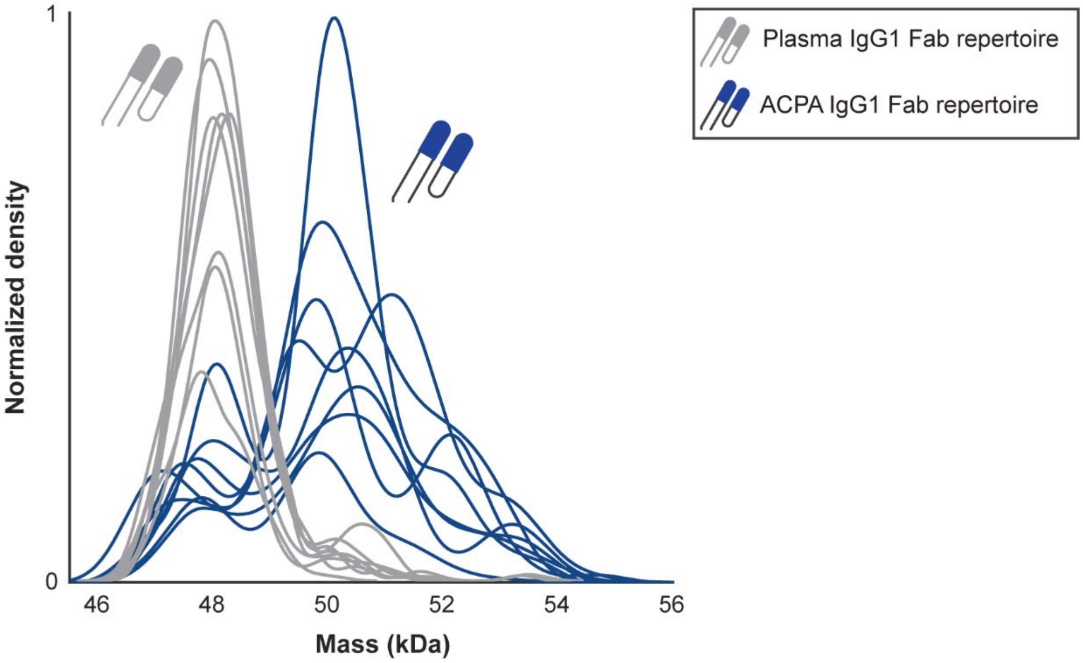

Supplementary Figure 7. Mass distribution of total plasma IgG1 and ACPA IgG1 Fab molecules detected for each individual in the cohort of RA patients studied (n=8). Distributions of total plasma IgG1 (grey) and ACPA IgG1 Fab masses (blue) are depicted as normalized kernel density estimation of all Fab molecules detected for the respective repertoire. The kernel density estimations were normalized per sample type.

a

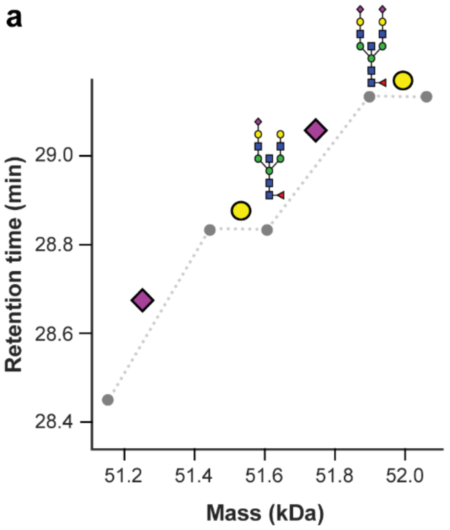

b

<table>
<tr><th></th><th></th><th>Mass addition ± SD (Da)</th><th>Mean RT shift | Max. RT shift (min)</th></tr>
<tr><td>◆</td><td>Sialic acid</td><td>291 ± 0.89</td><td>+0.35 | +0.45</td></tr>
<tr><td>●</td><td>Galactose</td><td>162 ± 0.99</td><td>+0.00 | +0.09</td></tr>
<tr><td>■</td><td>N-Acetylglucosamine</td><td>203 ± 0.50</td><td>+0.01 | +0.09</td></tr>
</table>

Supplementary Figure 8. Mass and retention time shifts detected for a Fab-glycosylated monoclonal ACPA IgG1. To determine the impact of glyco-heterogeneity on the obtained Fab profiles, a Fab-glycosylated monoclonal ACPA IgG1 was applied to total IgG1 Fab profiling, and mass and retention time shifts between the glycovariants of the generated monoclonal ACPA IgG1 Fab fragments were determined. The detected mass and retention time shifts were used as a starting point to identify Fab molecules in ACPA IgG1 Fab profiles that differed by individual monosaccharides. **(a)** Exemplary shifts in mass (x-axis) and retention time (y-axis) detected for monoclonal ACPA IgG1 glycovariants differing by individual monosaccharides. **(b)** Average mass and retention time (RT) shifts observed for the monoclonal ACPA IgG1. Mass shifts are indicated as mass addition with standard deviation (SD). RT shifts are indicated as mean and maximum shift. Of note, the retention time remained stable despite addition of an N-acetylglucosamine or galactose, whereas it increased upon addition of sialic acid.

Supplementary Table 1. Characteristics of the cohort studied. Anti-citrullinated protein antibody (ACPA) IgG levels were determined by in-house ACPA IgG ELISA as detailed in Methods. Treatment at time of sampling included MTX: methotrexate, Pred: prednisolone, HCQ: hydroxychloroquine, SSZ: sulfasalazine, azathioprine and Toci: tocilizumab as specified in the Table. DAS: disease activity score. ESR: erythrocyte sedimentation rate.

Patient	Age (years)	Sex (F/M)	Diagnosis	Duration of diagnosis (years)	ACPA status	ACPA IgG level (AU/mL)	DAS28	ESR	Treatment at time of sampling
**1**	61	F	RA	4	positive	2186	6.34	74	MTX 25 mg/wk, Pred 5 mg/d
**2**	62	F	RA	9	positive	>12800	2.79	2	MTX 15 mg/wk, Pred 7.5 mg/d
**3**	69	F	RA	16	positive	1822	3.76	29	MTX 12.5 mg/wk, HCQ 400 mg/d
**4**	84	M	RA	5	positive	3790	2.44	22	MTX 15 mg/wk, Pred 2.5 mg/d
**5**	58	M	RA	1	positive	2141	2.79	6	MTX 25 mg/wk
**6**	57	M	RA	26	positive	2504	4.04	48	MTX 20 mg/wk, SSZ 3 g/d, HCQ 400 mg/d, azathioprine 100 mg/d
**7**	54	M	RA	0	positive	1528	3.01	9	none
**8**	67	F	RA	24	positive	201	3.12	2	Toci 162 mg/wk, Pred 5 mg/d

Supplementary Table 2. Absolute number of ACPA IgG1 and total plasma IgG1 Fab molecules detected by LC-MS-based Fab profiling and relative contribution of the ten most abundant Fab molecules to the total ACPA and plasma IgG1 repertoires detected.

Patient	ACPA IgG1		Plasma IgG1	
	Fab molecules (#)	Contribution of Top 10 (%)	Fab molecules (#)	Contribution of Top 10 (%)
1	1019	20.6	401	25.7
2	1163	34.1	337	19.7
3	956	23.4	573	71.1
4	795	23.1	626	14.7
5	345	23.6	148	43.6
6	628	27.9	301	54.7
7	718	47.0	305	39.8
8	650	36.3	365	25.5

Supplementary Table 3. Total and ACPA IgG plasma concentration and relative abundance of ACPA IgG in total plasma IgG determined by ELISA. The ACPA IgG plasma concentration was determined by total IgG ELISA of purified ACPA eluates. The ACPA IgG quantity obtained per ACPA eluate was corrected for the volume of the respective ACPA eluate as well as the total plasma volume used for the respective ACPA purification.

Patient	Total IgG plasma concentration (mg/mL)	ACPA IgG plasma concentration (mg/mL)	ACPA IgG (% of total plasma IgG)
**1**	4.2	0.059	1.41
**2**	3.0	0.137	4.56
**3**	11.7	0.080	0.68
**4**	13.8	0.041	0.30
**5**	2.1	0.021	0.99
**6**	7.4	0.041	0.55
**7**	8.5	0.045	0.53
**8**	9.2	0.022	0.24

Supplementary Table 4. Monoclonal antibodies used throughout the study. The non-glycosylated monoclonal anti-TT IgG1 and ACPA IgG1 (7E4NG) as well as the Fab-glycosylated monoclonal ACPA IgG1 (7E4WT) were produced in-house as detailed in Methods. The monoclonal antibodies alemtuzumab and trastuzumab were kindly provided by Genmab, and Dietmar Reusch and Markus Haberger (Roche, Penzberg), respectively. The number of Fab glycans present and the theoretical mass of each monoclonal antibody is listed. The listed theoretical mass reflects the antibody protein mass without posttranslational glycosylation.

Monoclonal antibody	Source	Fab glycans present (#)	Theoretical mass (Da)
**Anti-TT IgG1**	In-house	0	48,693
**7E4NG**	In-house	0	46,772
**7E4WT**	In-house	2	46,785
**Alemtuzumab**	Genmab	0	47,859
**Trastuzumab**	Roche	0	47,500
