# Supplementary material for: Antigen-specific Fab profiling achieves molecular-resolution analysis of human autoantibody repertoires in rheumatoid arthritis
Source: Nat Commun. 2024 Apr 10;15:3114. doi: 10.1038/s41467-024-47337-x (PMC11006680; doi:10.1038/s41467-024-47337-x)
Supplement: Supplementary file 3 — Reporting Summary [file 41467_2024_47337_MOESM3_ESM.pdf]

nature portfolio

Corresponding author(s): Albert Bondt
Last updated by author(s): Mar 5, 2024

Reporting Summary

Nature Portfolio wishes to improve the reproducibility of the work that we publish. This form provides structure for consistency and transparency in reporting. For further information on Nature Portfolio policies, see our Editorial Policies and the Editorial Policy Checklist.

Statistics

For all statistical analyses, confirm that the following items are present in the figure legend, table legend, main text, or Methods section.

n/a	Confirmed	
☐	☒	The exact sample size (*n*) for each experimental group/condition, given as a discrete number and unit of measurement
☐	☒	A statement on whether measurements were taken from distinct samples or whether the same sample was measured repeatedly
☐	☒	The statistical test(s) used AND whether they are one- or two-sided<br>*Only common tests should be described solely by name; describe more complex techniques in the Methods section.*
☒	☐	A description of all covariates tested
☐	☒	A description of any assumptions or corrections, such as tests of normality and adjustment for multiple comparisons
☐	☒	A full description of the statistical parameters including central tendency (e.g. means) or other basic estimates (e.g. regression coefficient) AND variation (e.g. standard deviation) or associated estimates of uncertainty (e.g. confidence intervals)
☐	☒	For null hypothesis testing, the test statistic (e.g. *F*, *t*, *r*) with confidence intervals, effect sizes, degrees of freedom and *P* value noted<br>*Give P values as exact values whenever suitable.*
☒	☐	For Bayesian analysis, information on the choice of priors and Markov chain Monte Carlo settings
☒	☐	For hierarchical and complex designs, identification of the appropriate level for tests and full reporting of outcomes
☒	☐	Estimates of effect sizes (e.g. Cohen's *d*, Pearson's *r*), indicating how they were calculated

Our web collection on statistics for biologists contains articles on many of the points above.

Software and code

Policy information about availability of computer code

Data collection	BioPharmaFinder 3.2 (Thermo Scientific)
Data analysis	Data analysis scripts were written in Python 3.9.13 using libraries: Pandas 1.4.4, Numpy 1.21.5, Scipy 1.9.1, Matplotlib 3.5.2 and Seaborn 0.11.2.<br>ELISA quantifications were performed using the Microplate manager software MPM-6 (BioRad).<br>GraphPad Prism 9.3.1 was used for statistics.

For manuscripts utilizing custom algorithms or software that are central to the research but not yet described in published literature, software must be made available to editors and reviewers. We strongly encourage code deposition in a community repository (e.g. GitHub). See the Nature Portfolio guidelines for submitting code & software for further information.

Data

Policy information about availability of data

All manuscripts must include a data availability statement. This statement should provide the following information, where applicable:

- Accession codes, unique identifiers, or web links for publicly available datasets
- A description of any restrictions on data availability
- For clinical datasets or third party data, please ensure that the statement adheres to our policy

The raw spectra of the mass spectrometry data have been deposited to the MassIVE repository with identifier MSV000093196. Processed mass spectrometry data

underlying donut plots, heatmaps, dot plots as well as raw data underlying depicted chromatography traces and the background-subtracted raw ELISA data are provided in the source data file. The median mass of Fab molecules was determined based on the IMGT database (Lefranc M-P, et al. IMGT, the international ImMunoGeneTics database. Nucleic Acids Research 27, 209-212 (1999).)

Research involving human participants, their data, or biological material

Policy information about studies with human participants or human data. See also policy information about sex, gender (identity/presentation), and sexual orientation and race, ethnicity and racism.

Reporting on sex and gender	The study cohort was selected based on diagnosis, ACPA status and levels of ACPA autoantibodies. Due to the restricted size of the cohort, information on self-reported sex of each individual was collected but not used to stratify the observations. Although RA affects primarily women, currently available data do not indicate that the ACPA B cell response and/or characteristics of ACPA differ between male and female patients.
Reporting on race, ethnicity, or other socially relevant groupings	*Please specify the socially constructed or socially relevant categorization variable(s) used in your manuscript and explain why they were used. Please note that such variables should not be used as proxies for other socially constructed/relevant variables (for example, race or ethnicity should not be used as a proxy for socioeconomic status).*<br>*Provide clear definitions of the relevant terms used, how they were provided (by the participants/respondents, the researchers, or third parties), and the method(s) used to classify people into the different categories (e.g. self-report, census or administrative data, social media data, etc.)*<br>*Please provide details about how you controlled for confounding variables in your analyses.*
Population characteristics	Patients were all diagnosed with rheumatoid arthritis and their age ranged from 54 to 84 y.o.<br>There were 4 females and 4 males included.
Recruitment	Plasma used in this study was collected from patients visiting the outpatient clinic of the Rheumatology Department at the Leiden University Medical Center (LUMC). Patients recruited for the study were selected based on diagnosis, ACPA status and levels of ACPA autoantibodies. The latter was necessary solely to ensure that sufficient quantities of ACPA could be isolated for subsequent analysis. The study cohort includes patient plasma with ACPA IgG levels mostly >1500 AU/mL based on routine in-house testing. These levels are considered 'high'. ACPA IgG levels, however, are not considered to reflect clinical disease activity or other parameters relevant to the current study. The reported observations were consistent independent of the ACPA level of the particular individual.
Ethics oversight	Permission for conduct of the study was approved by the Ethical Review Board of the LUMC (protocols P13.171 and P17.151).

Note that full information on the approval of the study protocol must also be provided in the manuscript.

Field-specific reporting

Please select the one below that is the best fit for your research. If you are not sure, read the appropriate sections before making your selection.

☒ Life sciences ☐ Behavioural & social sciences ☐ Ecological, evolutionary & environmental sciences

For a reference copy of the document with all sections, see nature.com/documents/nr-reporting-summary-flat.pdf

Life sciences study design

All studies must disclose on these points even when the disclosure is negative.

Sample size	No sample size calculation was performed, sample size was based on sample availability and labor feasibility.
Data exclusions	One donor was excluded because an apparent matrix effect on retention times was observed in both the plasma ACPA repertoire and total repertoire measurements. Therefore no fair comparison of the repertoires was possible with the other donors.
Replication	To validate the approach, ACPA IgG1 Fab profiling was replicated for two patient plasma samples. Both replicates yielded highly similar results.
Randomization	No randomization was performed since only one experimental group was assessed. The quality of sample preparation and mass spectrometry measurements was monitored by ELISA and by internal as well as external controls, respectively.
Blinding	Since sample preparation deviated per sample type no blinding was applied.

Reporting for specific materials, systems and methods

We require information from authors about some types of materials, experimental systems and methods used in many studies. Here, indicate whether each material, system or method listed is relevant to your study. If you are not sure if a list item applies to your research, read the appropriate section before selecting a response.

Materials & experimental systems

n/a	Involved in the study
☐	☒ Antibodies
☐	☒ Eukaryotic cell lines
☒	☐ Palaeontology and archaeology
☒	☐ Animals and other organisms
☒	☐ Clinical data
☒	☐ Dual use research of concern
☒	☐ Plants

Methods

n/a	Involved in the study
☒	☐ ChIP-seq
☒	☐ Flow cytometry
☒	☐ MRI-based neuroimaging

Antibodies

Antibodies used

Trastuzumab, Roche, Penzberg, Germany, N/A. Alemtuzumab, Genmab, Utrecht, The Netherlands, N/A.
HRP-labelled rabbit anti-human IgG (DAKO, P0214)
goat anti-human IgG-Fc (Bethyl, A80-104)
HRP-labelled goat anti-human IgG (Bethyl, A80-104P)
Monoclonal ACPA and anti-TT were produced in-house at the LUMC.

Validation

Trastuzumab and Alemtuzumab were spiked into all samples and detected at masses and retention times expected. In-house produced monoclonal ACPA and anti-TT antibodies were produced using sequence-validated plasmids, assessed on SDS-PAGE and (antigen-specific) Fab profiling, which resulted in expected masses. The specificity of the in-house produced monoclonal antibodies was previously determined by ELISA (e.g. in Kissel et al., Annals of the Rheumatic Diseases, 2020).
The remaining antibodies used for ELISA (HRP-labelled rabbit anti-human IgG (DAKO, P0214); goat anti-human IgG-Fc (Bethyl, A80-104) and HRP-labelled goat anti-human IgG (Bethyl, A80-104P)) were validated within the study based on non-IgG, non-ACPA and non-TT IgG containing samples. Further validation of the goat anti-human IgG-Fc (Bethyl, A80-104) and HRP-labelled goat anti-human IgG (Bethyl, A80-104P) can be found on the manufacturer's website including 6 citations for goat anti-human IgG-Fc (Bethyl, A80-104) and >70 citations for HRP-labelled goat anti-human IgG (Bethyl, A80-104P).

Eukaryotic cell lines

Policy information about cell lines and Sex and Gender in Research

Cell line source(s)

In-house produced monoclonal antibodies were produced in FreestyleTM 293-F cells (Gibco).

Authentication

The cell line was not authenticated, but purchased from the supplier and phenotypically behaved authentic.

Mycoplasma contamination

The cell line was tested negative for mycoplasma contamination.

Commonly misidentified lines
(See ICLAC register)

No commonly misidentified cell lines were used in this study.

Plants

Seed stocks

Report on the source of all seed stocks or other plant material used. If applicable, state the seed stock centre and catalogue number. If plant specimens were collected from the field, describe the collection location, date and sampling procedures.

Novel plant genotypes

Describe the methods by which all novel plant genotypes were produced. This includes those generated by transgenic approaches, gene editing, chemical/radiation-based mutagenesis and hybridization. For transgenic lines, describe the transformation method, the number of independent lines analyzed and the generation upon which experiments were performed. For gene-edited lines, describe the editor used, the endogenous sequence targeted for editing, the targeting guide RNA sequence (if applicable) and how the editor was applied.

Authentication

Describe any authentication procedures for each seed stock used or novel genotype generated. Describe any experiments used to assess the effect of a mutation and, where applicable, how potential secondary effects (e.g. second site T-DNA insertions, mosiacism, off-target gene editing) were examined.
